# The Use of Imaging in the Prediction and Assessment of Cancer Treatment Toxicity

**DOI:** 10.3390/diagnostics7030043

**Published:** 2017-07-20

**Authors:** Hossein Jadvar

**Affiliations:** Division of Nuclear Medicine, Department of Radiology, Keck School of Medicine, University of Southern California, Los Angeles, CA 90033, USA; jadvar@med.usc.edu; Tel.: +1-323-442-1107; Fax: +1-323-442-3253

**Keywords:** toxicity, therapy, cancer, imaging

## Abstract

Multimodal imaging is commonly used in the management of patients with cancer. Imaging plays pivotal roles in the diagnosis, initial staging, treatment response assessment, restaging after treatment and the prognosis of many cancers. Indeed, it is difficult to imagine modern precision cancer care without the use of multimodal molecular imaging, which is advancing at a rapid pace with innovative developments in imaging sciences and an improved understanding of the complex biology of cancer. Cancer therapy often leads to undesirable toxicity, which can range from an asymptomatic subclinical state to severe end organ damage and even death. Imaging is helpful in the portrayal of the unwanted effects of cancer therapy and may assist with optimal clinical decision-making, clinical management, and overall improvements in the outcomes and quality of life for patients.

## 1. Introduction

Cancer treatment has evolved considerably, with significant improvements in various outcome measures. Some malignancies may be amenable to cure, while some can be managed as a chronic disease. These achievements are fundamentally based on the ever-growing advances in our understanding of the complex biology and spatiotemporal heterogeneity of cancer. Innovations in multimodal imaging have also provided unprecedented opportunities to contribute to this quest. Imaging has become a major component of comprehensive cancer care and may be used for diagnosis, staging, assessing treatment response, restaging after therapy, and prognosis.

Cancer treatments are varied and are evolving towards precision therapy based on the underlying molecular profile of tumors. Most treatments are associated with at least some level of undesired toxicity, which may be due to a direct effect on non-tumor tissue or the body’s reaction to the treatment’s direct damage of tumor cells. Imaging can play a major role in the assessment of anticipated and, occasionally, the unanticipated toxicity of cancer treatment. Treatment-induced toxicity is reported on a grading scale from one to five [1]. Grade one toxicity denotes asymptomatic or mildly symptomatic adverse events which may be observed on imaging and often do not lead to the need for intervention. With an increasing grade score, the severity of toxicity increases; to the extent that grade five denotes death. Typically, grade two toxicity (a moderately adverse event) may lead to an intervention, including a decrease in drug doses or the use of steroids [2]. In this article, we briefly review the use of imaging in the assessment of cancer treatment toxicity—organized by organ systems—providing a concise guide to the published literature on this topic. A comprehensive glossary of imaging features for various cancer treatment-related conditions is not the intention of this narrative review. The interested reader may refer to the relevant specified references for such details.

## 2. Neurological Toxicity

Cancer therapy-associated neurotoxicity can occur in patients regardless of the site and type of tumor [3]. Perry et al. have reviewed the literature on cancer therapy-associated neuropathology [4]. Chemotherapy can occasionally lead to significant neurotoxicity; for example, platinum-based drugs cause peripheral neuropathy by damaging sensory neurons within the dorsal root ganglia. Predicting the occurrence and severity of neurotoxicity remains challenging [5]. The damage caused by cancer therapy may have a variable onset (acute, delayed) and include direct cellular toxicity, changes in cellular function, and other adaptations such as inflammation that can indirectly cause injury [6]. Manifestations of neurotoxicity can be varied, including alterations in attention, cognitive impairment, psychiatric events, diminished executive functions, cerebrovascular complications, diffuse brain atrophy, and posterior reversible encephalopathy syndrome (PRES) [7]. PRES may be associated with a variety of immunosuppressive therapies and other agents such as cisplatin, rituximab, and bevacizumab. There may be multiple predisposing host risk factors that can contribute to the development of neurotoxicity, including patient’s age, genetic background, and their predisposition to idiosyncratic reactions [4]. The relevant biological factors may include polymorphisms in folate metabolizing enzymes and apolipoprotein E, as well as those in blood-brain barrier transporter genes [8].

Imaging is often used not only to assess the response to therapy and to differentiate between radiation necrosis and residual or recurrent tumors but also to detect and characterize potential chemotherapy-associated toxicity [9,10]. Moreover, a combination of pre, during and post-chemotherapy imaging assessments of relevant biomarkers may facilitate the querying process of the underlying mechanisms that are involved in therapy-induced neurotoxicity. The use and limitations of various imaging modalities in the assessment of cancer treatment-related neurotoxicity have previously been reviewed [11,12,13]. Generally, anatomically-based imaging modalities, particularly magnetic resonance (MR) imaging, can be helpful in the assessment of inflammation, edema, atrophy, necrosis, gliosis, hemorrhage, ischemia, etc. For example, Futterer et al. showed that MR diffusion abnormalities might be seen in the corpus callosum of patients receiving bevacizumab therapy for malignant brain tumors [14]. In PRES, there are often posterior brain subcortical white and gray matter lesions on fluid-attenuated inversion recovery (FLAIR) and T2-weighted sequences [10]. There are relatively few scintigraphic studies dedicated to the imaging assessment of therapy-associated neurotoxicity. However, single photon computed tomography (SPECT) and positron emission tomography (PET) with relevant radiotracers could assess perfusion and metabolism and various biomarkers—including conditions such as cognition—which may become altered during cancer therapy.

## 3. Pulmonary Toxicity

The lung is a common site of cancer therapy-related acute and chronic toxicity caused by radiotherapy and several anticancer drugs such as methotrexate, paclitaxel, docetaxel, and gemcitabine. Radiation therapy (RT) is often employed in the treatment of lung cancer. While treatment planning is optimized to limit non-target radiation, some damage may occur along the path of the radiation beam. Post-RT lung density changes on computed tomography (CT) and symptomatic radiation pneumonitis have been found to be associated with RT techniques, total doses as low as 16–30 Gy, and increasing age [15]. Farr et al. studied the potential use of perfusion SPECT in predicting the risk of RT in combination with standard CT-based dose-volume parameters in patients with non-small-cell lung cancer who were undergoing radiotherapy [16]. Perfusion SPECT could be used to improve radiotherapy planning and reduce pulmonary radiotoxicity. Earlier studies with ^99m^Tc-DTPA aerosol inhalation planar lung scintigraphy had shown that there was a significantly shorter clearance time in patients with chemotherapy-induced pulmonary damage compared with their pretreatment state or to those who did not receive chemotherapy [17,18]. Petit et al. hypothesized that pretreatment pulmonary inflammation renders the lung more susceptible to radiotoxicity [19]. In a retrospective study of 101 patients with non-small-cell lung cancer who were treated with chemo-radiation therapy, ^18^F-fluorodeoxyglucose (FDG) PET/CT was performed to assess the relationship between radiation-induced lung injury and pretreatment increased lung density, on CT, and pretreatment pulmonary FDG uptake, on PET. The risk of lung radiotoxicity was increased in those lung segments that showed a high pretreatment pulmonary FDG uptake, suggesting that the risk of radiation injury may be decreased if the areas of pulmonary hypermetabolism subtended by the radiation port can be minimized [20]. MacManus and colleagues determined that for every one-level increase in a defined visual scoring of lung FDG uptake, the risk of radiation pneumonitis increased by 40% [21].

**Figure 1:**
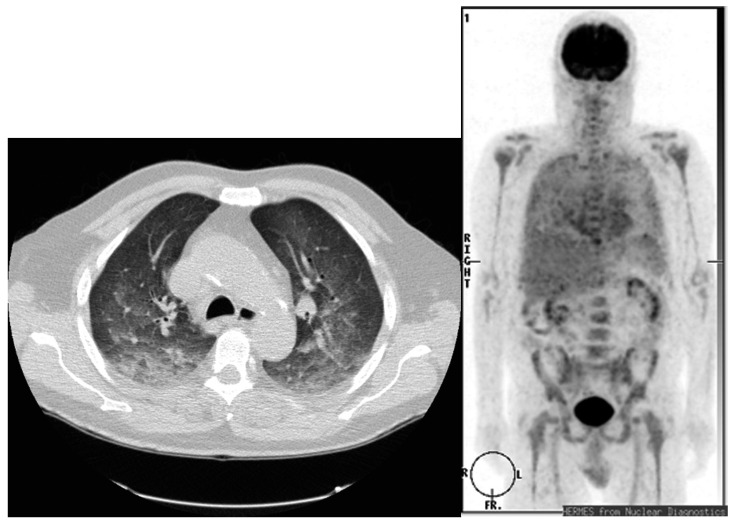
Pulmonary toxicity in a patient with chronic myelomonocytic leukemia treated with etoposide. A computer tomography (CT) scan (**left panel**) shows diffuse ground glass and reticular opacities in bilateral posterior pulmonary segments. A coronal ^18^F-fluorodeoxyglucose (FDG) positron emission tomography (PET) scan (**right panel**) demonstrates bilateral diffuse pulmonary uptake (Used with permission from [29]).

In another investigation, FDG PET/CT was employed to evaluate late pulmonary toxicity which was induced by rituximab-containing chemotherapy in patients with non-Hodgkin lymphoma [22]. Asymptomatic subpleural pulmonary interstitial abnormalities were noted on FDG PET/CT, with mild to moderate hyper-metabolism at 1 to 3 months post treatment. The authors warned that this pattern should not be mistaken for lymphoma recurrence. A similar observation has been reported in a larger group of 460 patients with lymphoma who underwent chemotherapy and serial FDG PET/CT scans [23]. Diffuse ground-glass opacities with peripheral-dominant pulmonary FDG uptake was noted in asymptomatic patients. In patients exposed to cyclophosphamide, bleomycin, or everolimus, a range of findings may be seen on CT, while diffuse high bilateral pulmonary FDG uptake may be present on PET [24,25,26,27,28,29] (Figure 1). Other chemotherapy agents, such as gemcitabine, can also cause a diversity of pulmonary toxicity ranging from mild dyspnea to severe pulmonary fibrosis and acute respiratory distress syndrome [30]. The CT findings are nonspecific and typically demonstrate ground-glass opacities with or without smoothly thickened septal lines (reticular) and, in the most severe cases, a honeycomb fibrotic pattern with possible diffuse alveolar infiltrates [31]. Comprehensive reviews of chest CT findings in pulmonary complications (e.g., bronchiolitis obliterans organizing pneumonia, nonspecific interstitial pneumonia, eosinophilic pneumonia, obliterative bronchiolitis, diffuse alveolitis, etc.) from cancer treatment have been published [32,33,34].

## 4. Cardiac Toxicity

**Figure 2:**
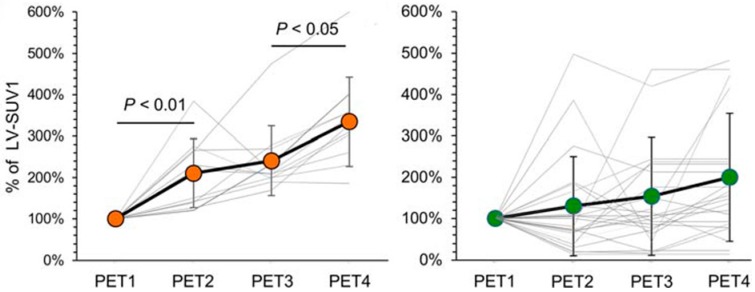
Myocardial FDG uptake significantly increases in HD patients with late treatment-related cardiac abnormalities, defined as electrocardiographic or echocardiographic abnormalities (**left panel**) compared to those patients without late therapy-induced cardiotoxicity (**right panel**). FDG PET/CT scans: the baseline at staging (PET1); a negative interim scan during chemotherapy (PET2), a negative scan after the completion of adriamycin, bleomycin, vinblastine, and dacarbazine (ABVD) chemotherapy at 4–6 weeks post-therapy (PET3) and a negative six-month follow-up scan (PET4) (Used with permission from [51]).

The cardiovascular system is often affected by various cancer drug therapies (e.g., anthracyclines, monoclonal antibodies, fluoropyrimidines, taxanes, alkylating agents, vinka alkaloids, and angiogenesis inhibitors) [35,36,37]. Cardiac toxicity can become chronic, with significant irreversible morbidity and even mortality [38,39,40]. The diagnostic imaging techniques that can be used to assess cardiac toxicity include echocardiography, scintigraphy, CT, and MRI [41,42,43,44]. However, the early prediction of cardiac injury remains challenging; easier prediction would be desirable in order to accordingly adapt chemotherapy for optimal clinical management. The majority of optimal imaging modalities and measurement parameters are unsettled [45]. The measurement of left ventricular ejection fraction (LVEF) with either echocardiography or multiple-gated acquisition (MUGA) radionuclide angiography is currently the most common approach used [46,47]. Cancer treatment-related cardiotoxicity is defined as a >5% reduction in LVEF to <55% with heart failure symptoms, or a >10% reduction to <55% in asymptomatic patients [48]. However, both echocardiography and MUGA scintigraphy are relatively insensitive to early cardiac injury and small changes in LVEF. Aiken et al. reviewed the specific case for the use of MUGA scintigraphy in the assessment of doxorubicin-induced cardiotoxicity [49]. With regards to a radiation-induced decline in myocardial perfusion, Zellars and colleagues, using SPECT, have shown that an active breathing coordinator (which enables radiation-delivery when the chest wall is farther from the heart and, hence, less cardiac radiation exposure) does not prevent radiation-induced cardiac hypo-perfusion defects [50]. More recently, Italian researchers have investigated whether serial FDG PET/CT predicts doxorubicin cardiotoxicity [51]. This combined preclinical mice and retrospective clinical human (in patients with Hodgkin’s lymphoma (HD) treated with an adriamycin, bleomycin, vinblastine, and dacarbazine (ABVD) regimen) study showed that there were doxorubicin dose-dependent increases in left ventricular glucose consumption (LV-MRGlu), particularly in the presence of low baseline LV FDG uptake. The authors concluded that low myocardial FDG uptake prior to the initiation of doxorubicin chemotherapy in HD patients might predict the development of chemotherapy-induced cardiotoxicity (Figure 2).

Cardiac MRI is ideal in characterizing myocardial tissue and assessing chamber ventricular volume and function [52,53]. It has the advantages of lacking ionizing radiation and a high soft tissue contrast but has limitations with regards to its access, availability, and cost. The interested reader is referred to an excellent systematic review by Thavendiranathan and colleagues on the use of cardiac MRI in the assessment of cancer treatment-related toxicity, with specific sections focusing on the detection of early cardiac injury, the identification of short-term cardiotoxicity (<1 year of treatment), the detection of the late effect of therapy (>1 year after treatment), and the monitoring of the response to cardioprotective therapy [54]. This systematic review revealed that MRI evidence of myocardial inflammation and edema may portray the earliest signs of cancer treatment-related cardiotoxicity and the potential ensuing ventricular dysfunction. The authors suggest further investigations to decipher whether the higher cost of cardiac MRI can be adequately balanced by the ability to identify a higher-risk group who can benefit from preemptive targeted cardiac therapy, leading to a reduction in cardiac morbidity and a favorable cost-benefit ratio.

Konski et al. retrospectively evaluated 102 patients with esophageal cancer who were treated with chemo-radiotherapy and developed symptomatic cardiotoxicity [55]. Changes in FDG myocardial uptake as measured by standardized uptake value (SUV) did not correlate with cardiac toxicity. However, it must be noted that the determination of a potential relation in myocardial metabolism and a chemo-radiotherapy effect may be challenging, as normal myocardial FDG uptake is quite variable even with prolonged fasting preparation, while isolating physiologic metabolic variability from systemic drug-induced metabolic changes can be difficult [56]. Moreover, hyper-metabolism around the heart may reflect chemotherapy-induced pericarditis [57].

In summary, while the current, relatively simple imaging methods for determining gross left ventricular function changes (as reflected by LVEF) have been helpful in clinical decision-making, additional investigations into the multimodal imaging assessment of the earliest signs of treatment-induced myocardial damage may provide opportunities for adaptive treatments that optimize therapy efficacy while minimizing adverse cardiac events in a cost-effective manner for the large cohort of cancer patients who receive cardio-toxic treatments.

## 5. Hepatic and Gastrointestinal Tract Toxicity

**Figure 3:**
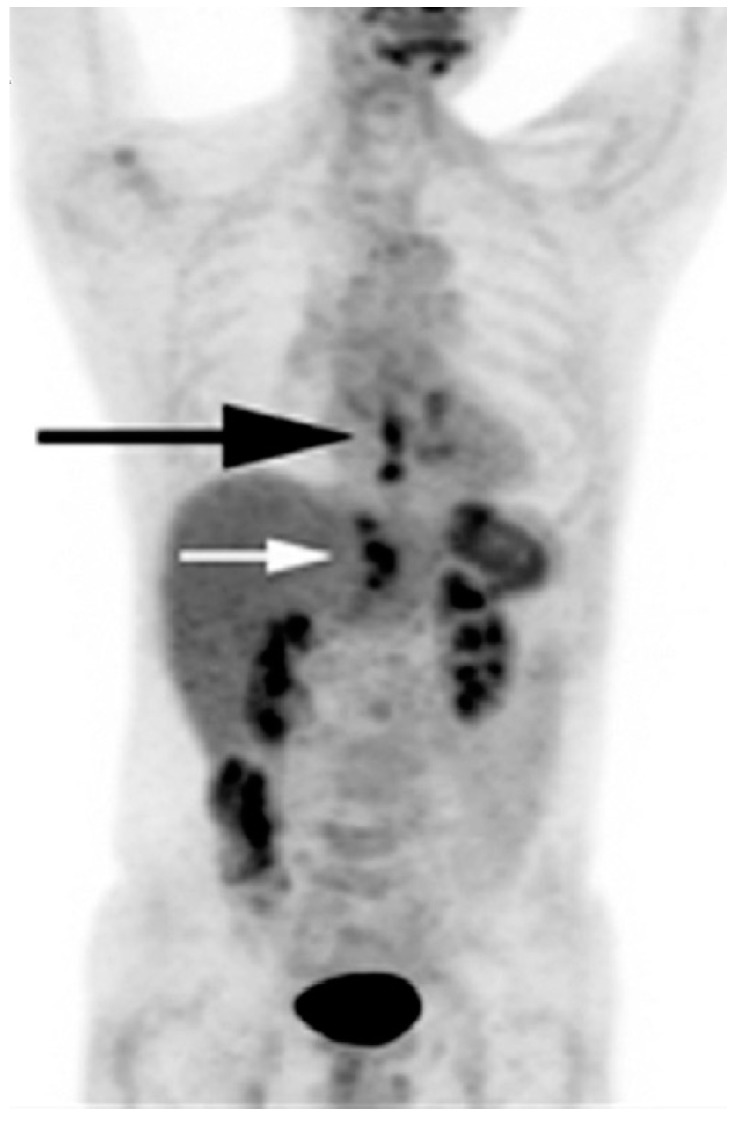
Hepatic radiation injury in a patient who underwent radiotherapy for distal esophageal cancer. The coronal maximum projection image (FDG PET image) shows residual esophageal tumor hypermetabolism (SUVmax = 3.4) (black arrow) and hypermetabolic areas (SUVmax = 3.5) in liver segments I, II, and III (white arrow). (Used with permission from [61]). SUV, standardized uptake value.

The liver, as the main physiological detoxifying organ, is another common site of cancer treatment toxicity [58,59]. A number of anticancer agents, such as 5-fluorouraci, leucovorin, bevacizumab, and pazopanib, may lead to hepatic steatosis. Hepatic steatosis is detected on unenhanced CT as diffuse fatty change, with a decline in hepatic attenuation by 10–25 Hounsfield Units (HU) below that in the spleen [60]. While simple hepatic steatosis may remain asymptomatic, an evolution to and development of steatohepatitis may lead to a decline in hepatic function and regeneration [31]. Radiation therapy to nearby organs may also result in hepatic injury [61] (Figure 3).

Selective intra-arterial delivery of chemotherapy or radioactive microsphere therapy has been shown to be safe and effective in a number of clinical settings, including unresectable hepatocellular carcinoma, unresectable or recurrent cholangiocarcinoma, and liver dominant metastases from colorectal cancer, breast cancer, renal cell carcinoma, pancreatic cancer, melanoma, and neuroendocrine tumors [62,63]. The therapy procedure is preceded by angiographic mapping, which includes ^99m^Tc-MAA hepatic perfusion delineation, pulmonary shunt calculation and coil embolization of aberrant or collateral vessels as needed to limit the exposure of and toxicity to non-tumor tissues from the therapy agent [64]. The interested reader is referred to comprehensive reviews of the side effects of intra-arterial radio-embolization [65]. Radiotoxicity is related to the radiation-induced toxicity (inflammation, ulceration, necrosis, abscess, and stricture) of the unintended exposed organs or tissues (e.g., stomach, duodenum, gallbladder, normal liver, pancreas, etc.). Atassi and colleagues provide an excellent review of the multimodality imaging findings for the recognition of potential complications and the assessment of the therapy response to radio-embolization [66].

Radiation therapy for lung cancer may result in esophageal injury. Symptomatic esophagitis may be predicted with the level of FDG uptake in the esophagus [67,68]. Similarly, the rest of the gastrointestinal tract may be affected, with signs such as gastritis, enteritis, colitis, pneumatosis intestinalis, bowel ischemia, infarction, hemorrhage and perforation [69,70,71,72,73]. Torrisi and colleagues provide an excellent review of the typical morphological changes, which may be seen on CT, associated with these conditions [31]. Scintigraphy may also be helpful for the evaluation of gastrointestinal bleeding and abdominal infection. Given that, on PET with FDG, the bowel shows variable uptake, which may also be affected by some medications (e.g., higher bowel FDG uptake with metformin therapy for diabetes mellitus), the assessment of enteric complications solely based on metabolic information may have limited use. However, indicators on the accompanied CT may be helpful (e.g., mesenteric fat strand lines adjacent to the bowel, fluid collections, bowel wall thickening, etc.). For example, intense small or large bowel FDG uptake with the aforementioned secondary abnormal signs on CT hints at enterocolitis. Chemotherapy is often associated with neutropenia, which may lead to neutropenic enterocolitis [74].

## 6. Urinary System Toxicity

Renal toxicity is a relatively common toxicity caused by chemotherapy (e.g., cisplatin, methotrexate) [75]. The typical signs include diminished renal function with a rising serum creatinine level, a decreased urine output, blood electrolyte or other metabolite derangements and the potential development of nephrotic syndrome. Multimodality imaging (e.g., ultrasonography, CT, MRI, scintigraphy) can be helpful for the assessment of renal changes in morphology and function that may have resulted from therapy-induced injury. Clearly, in patients with renal toxicity from systemic therapy, the iodinated contrast agents for CT studies or the gadolinium-based agents for MRI studies may only be used with caution, or not at all, in order to avoid compounding the harm to the kidneys. Good hydration is helpful in renal protection. In certain treatments, renal protection may be archived with the preemptive use of appropriate agents. Radiation nephropathy has been described in some patients who undergo peptide receptor radionuclide therapy (PRRT). The radiolabeled somatostatin analogs localize in proximal tubular cells. Methods that can interfere with the reabsorption pathway may aid in renal protection. Rolleman et al. provide a summary of the potential mechanisms for renal injury and the methods for renal protection in PRRT [76]. These methods include an infusion of amino acids (e.g., a mixture of lysine and arginine) to reduce renal reabsorption, other experimental approaches, such as the design and development of new peptides with a higher affinity and specificity for somatostatin receptors, and the use of radio-protective drugs. In the case of radiolabeled prostate specific membrane antigen (PSMA) targeted therapy in patients with metastatic castrate-resistant prostate cancer, the application of PSMA inhibitors such as 2-(phosphonomethyl)pentanedioic acid (PMPA) has been found to reduce off-target radiation to the kidneys [77]. The bladder may also be affected by chemotherapy (e.g., cyclophosphamide) toxicity, typically in form of cystitis, which may become hemorrhagic. Cross-sectional imaging findings are generally not specific, but may display diffuse, nodular, or combination diffuse-nodular bladder wall thickening.

## 7. Hematopoietic Toxicity

Bone marrow toxicity is a common occurrence after chemo-radiation therapy. Imaging-based prediction of the extent and severity of marrow suppression can be helpful in assessing and managing potential acute and late hematological toxicity. Metabolic imaging with PET is useful in assessing the effect of treatment on bone marrow and provides a possible platform for predicting marrow response after treatment [78,79,80]. A Tibetan mini-pig animal model study showed that FDG PET may be helpful in assessing absorbed radiation doses to the bone marrow and predicting the survival outcome [81]. McGuire et al. correlated the change in the uptake of the cellular proliferation PET biomarker, ^18^F-fluorothymidine (FLT), in the bone marrow of patients undergoing chemo-radiation therapy for pelvic cancer [82]. Radiation doses of 4 Gy after 1–2 weeks of therapy caused about a 50% decrease in FLT uptake, which was reflective of the decline in normal proliferating marrow cells. Interestingly, FLT uptake in marrow which was exposed to >35 Gy radiation was about 19% greater at 1 month after therapy than at 1 year after therapy, suggestive of chronic therapy-induced bone marrow suppression. In the case of PRRT for neuroendocrine tumors, it has been shown that hematological toxicity is not related to splenic radiation [83].

## 8. Skin Toxicity

**Figure 4:**
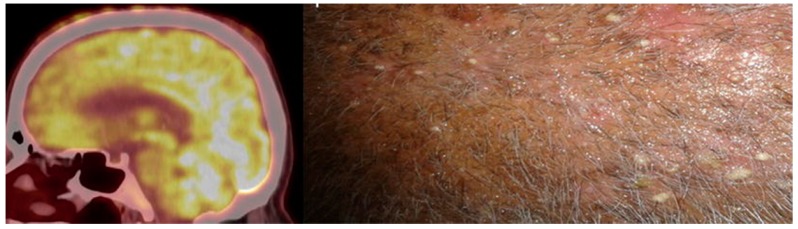
Cutaneous toxicity in a patient with metastatic lung cancer treated with erlotinib. A fused sagittal FDG PET-CT image of the skull shows multiple focal areas of FDG uptake in the scalp (**left panel**) that corresponded to pustular nodules, as seen on the photograph (**right panel**). (Used with permission from [84]).

Cancer treatments may lead to cutaneous toxicity. Kumar et al. describes a case report of patients with metastatic non-small-cell lung cancer undergoing erlotinib (a reversible epidermal growth factor receptor kinase inhibitor) who developed skin toxicity in the form of pustular skin nodules that demonstrated hyper-metabolism on FDG PET/CT [84] (Figure 4). Most skin nodules typically appear during the first week of treatment and disappear after the discontinuation of erlotinib, which can be useful in a differential diagnosis from skin metastases. Another case report showed similar findings for cutaneous nodular hyper-metabolism with FDG PET/CT in a patient with erythema nodosum-like panniculitis and in a patient with advanced melanoma treated with dabrafenib (a BRAF inhibitor) and trametinib (a MEK inhibitor) [85].

## 9. Conclusions

The treatment of cancer leads to an unwanted toxicity. The extent and severity of the adverse events may limit the type, dosing amount, and the number of therapy cycles, and occasionally may lead to the termination of treatment. Understanding the underlying mechanisms that are involved with various cancer therapy regimens can help in optimizing the effectiveness of the treatment while reducing any unwanted side effects. While some treatment toxicities may be asymptomatic, others may lead to irreversible end organ damage or even death. Imaging can play an important role in the care of cancer patients, not only for the assessment of the state of tumors and their response to therapy, but also in order to decipher the radiographic and scintigraphic signs of treatment toxicity.
